# Ground State Properties of the Wide Band Gap Semiconductor Beryllium Sulfide (BeS)

**DOI:** 10.3390/ma14206128

**Published:** 2021-10-15

**Authors:** Blaise A. Ayirizia, Janee’ S. Brumfield, Yuriy Malozovsky, Diola Bagayoko

**Affiliations:** Department of Mathematics and Physics, Southern University and A&M College, Baton Rouge, LA 70813, USA; janee_brumfield_00@subr.edu (J.S.B.); yuriy_malozovsky@subr.edu (Y.M.)

**Keywords:** zinc-blende structure, local density approximation, energy minimization, electronic energies and related properties

## Abstract

We report the results from self-consistent calculations of electronic, transport, and bulk properties of beryllium sulfide (BeS) in the zinc-blende phase, and employed an ab-initio local density approximation (LDA) potential and the linear combination of atomic orbitals (LCAO). We obtained the ground state properties of zb-BeS with the Bagayoko, Zhao, and Williams (BZW) computational method, as enhanced by Ekuma and Franklin (BZW-EF). Our findings include the electronic energy bands, the total (DOS) and partial (pDOS) densities of states, electron and hole effective masses, the equilibrium lattice constant, and the bulk modulus. The calculated band structure clearly shows that zb-BeS has an indirect energy band gap of 5.436 eV, from Γ to a point between Γ and X, for an experimental lattice constant of 4.863 Å. This is in excellent agreement with the experiment, unlike the findings of more than 15 previous density functional theory (DFT) calculations that did not perform the generalized minimization of the energy functional, required by the second DFT theorem, which is inherent to the implementation of our BZW-EF method.

## 1. Introduction

Group II–IV compounds have been widely studied in light of the growing demand for potentially good semiconductors for various electrical and optical devices. BeS is an interesting material with high hardness; it belongs to the beryllium chalcogenides family and crystallizes in the zinc-blende structure under normal condition. It has potential applications in blue-green laser diodes and light-emitting diodes. The material can be grown on various substrates by molecular beam epitaxy [1,2]. The toxicity of zb-BeS has partly resulted in the dearth of experimental results on its many properties. Muoz et al. [3] used first-principles calculations to show that BeS undergoes a phase transition to the nickel arsenide (NiAs) structure under high pressure. The band gaps of Be compounds are experimentally reported to range between 2.7 eV and 5.5 eV [4]. *Ab-initio* pseudopotential calculations [5] with the local density approximation (LDA) and the generalized gradient approximation (GGA) obtained an indirect band gap of 2.911 eV and 3.041 eV, respectively. The work of Gonzalez-Diaz et al. [6] employed the first-principles pseudopotential plane wave method and the Cerperly Alder form of the local density approximation potential, where they found an indirect band gap of 2.75 eV for zb-BeS. Benosman et al. [7] used the FP- LAPW method and a local density approximation for exchange and correlation potential to study the structural and electronic properties of BeS. They reported a band gap of 2.847 eV. Table 1 shows the findings from over 20 previous DFT calculations using ab-initio LDA or generalized gradient approximation (GGA) potentials. The 11 LDA calculations reported gaps in the 2.38 eV to 4.17 eV range. The nine (9) GGA computations found band gaps in the 3.041 eV to 4.241 eV range. These calculations not only disagree among themselves, but also with an experiment that produced a gap of 5.5 eV. The previous DFT calculations uniformly underestimated the band gap. Our motivation for this work stems from these disagreements and potential applications of zb-BeS. The results obtained with ad-hoc DFT potentials vary with the adjustable parameters germane to the construction of these potentials; consequently, we do not discuss here the calculations with these potentials due to their lack of predictive capabilities. Even though the Green function and dressed Coulomb approximation (GW) is beyond density functional theory, results from this approach, as per the content of the table, are also underestimates of the measured band gap of zb-BeS.

Engel and Vosko generalized-gradient approximation (EV-GGA) and screened exchange local density approximation (Sx-LDA).

This motivation is underscored by the fact that an accurate value of the band gap is necessary for producing correct, theoretical descriptions of electronic, optical, dielectric, transport, and related properties of semiconductors and insulators. Our computational method, described below, performs a generalized minimization of the energy functional to reach, verifiably, the ground state without using over-complete basis sets. In doing so, its results possess the full, physical content of DFT and generally agrees with the corresponding, experimental ones.

## 2. Computational Method and Related Details

We employed the Ceperley and Alder’s [20] local density approximation potential, which was parameterized by Vosko et al. [21]. Based on DFT, it fully minimized the energy functional with the Bagayoko, Zhao, and Williams (BZW) method [22,23,24], as enhanced by Ekuma and Franklin (BZW-EF) [25,26,27,28], while implementing the linear combination of atomic orbitals (LCAO). We employed a program package developed at the Ames Laboratory of the U.S. Department of Energy, Ames, Iowa [29,30].

We begin our self-consistent calculations with a small basis set that can account for all the electrons in the system under study. Calculation II uses the basis set of calculation I plus one orbital representing an excited state. The occupied energies of calculation I and calculation II were compared, graphically and numerically, with the Fermi energy set to zero. Some occupied energies from calculation II were lower than their values from calculation I. This lowering of occupied energies indicates that the basis set of calculation I is not complete for the description of the ground state. If it were, no augmented basis set would have lowered the occupied energies that have reached their absolute minima (i.e., the ground state). We have no proof that calculation II reached the ground state, so we continued the process of augmenting the basis set and of performing successive, self-consistent calculations. When three (3) consecutive calculations produced the same occupied energies, within our computational uncertainty of 5 meV, this is the proof that these energies have reached their absolute minima (i.e., the ground state). These three (3) calculations constitute the rigorous criteria for ending the process.

The first of the above referenced three (3) consecutive calculations, with the smallest basis set, provides the true DFT description of the material. The occupied energies of these calculations are unaffected (i.e., they do not change), however, the unoccupied energies from these calculations are either lower than or equal to their corresponding values produced with the *optimal basis set* [25,27,31]. In the discussion section, we address this extra-lowering of some unoccupied energies while the occupied ones remain unchanged. The most important thing to emphasize here is that unoccupied energy values that are lower than their corresponding values obtained with the optimal basis no longer belong to the spectrum of the Hamiltonian, a unique functional of the electronic density [25]. Another way of proving this fact follows. The charge density and the potential did no change from the first of the three (3) calculations to the last. Hence, the Hamiltonian, a unique function of the density, does not change either. Therefore, any unoccupied eigenvalues from the second or third of the three (3) calculations that is lowered than it corresponding value from the first calculation is not due to a physical interaction. Bagayoko [25] explained this fact and ascribed the lowering of these unoccupied energies to a mathematical artifact stemming from the Rayleigh theorem for eigenvalues. The theorem asserts the lowering of some eigenvalues with the increase in the dimension of the Hamiltonian matrix [30].

The computational details that permit the replication of this work are as follows: BeS crystallizes in the cubic 216 (*Fm-*$\bar{4}$*m*) space group where the positions of the atoms of Be and S are (0,0,0) and (1/4,1/4,1/4), respectively, with an experimental lattice constant of 4.86 Å [32]. We began by performing self-consistent ab-initio calculations for the ionic species Be^2+^ and S^2−^, and we employed a set of even-tempered Gaussian exponents to expand the radial components of the atomic wave functions in terms of Gaussian functions. The s and p orbitals for the ionic species (Be^2+^ and S^2−^) were described using 16 and 22 even tempered Gaussian functions, respectively. For Be^2+^, the maximum and minimum exponents utilized were 0.9 × 10^5^ and 0.24, respectively, whereas for S^2−^, they were 0.24 × 10^6^ and 0.135, respectively. After 60 iterations, for 81 k-points in the irreducible Broullouin zone, self-consistency was achieved when the difference in the potential between two consecutive iterations was less than or equal to 10^−5^.

## 3. Results

### 3.1. Electronic Properties

The valence orbitals in the basis set for the successive, self-consistent calculations with the BZW-EF computational method are listed in Table 2, along with the resulting band gaps. The occupied bands from calculations III–V are perfectly superimposed. Hence, they are the three (3) calculations producing the same, occupied energies that have reached their absolute minima (i.e., the ground state). Calculation III is therefore the one providing the true DFT description of zb-BeS. The calculated indirect band gap from Γ to a point between Γ and X was 5.436 eV. The minimum in the conduction band occurred along the Γ-X line, near the X point.

**Figure 1:**
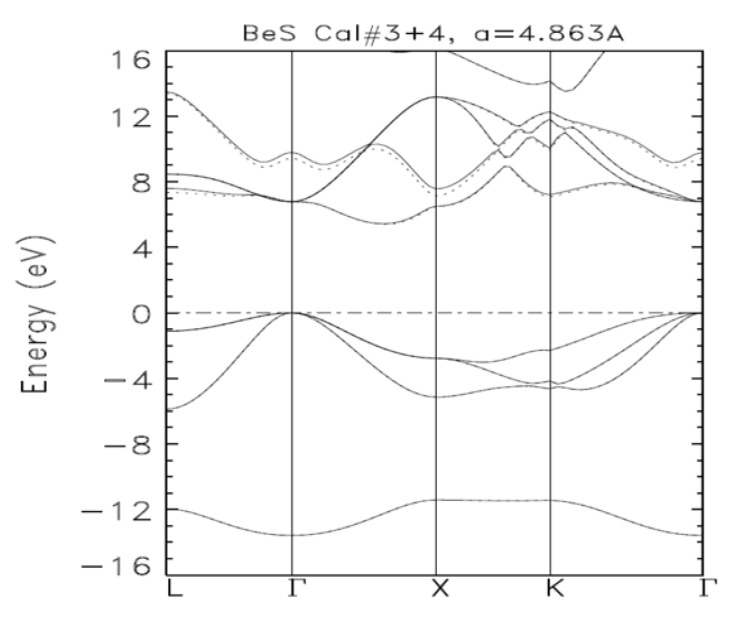
The electronic band structures of zb-BeS for a room temperature experimental lattice constant of 4.863 Å as obtained from calculations III (solid lines) and IV (dashed lines). Zero on the vertical axis denotes the position of the Fermi energy.

Figure 1 shows the electronic band structures of zb-BeS obtained from calculations III and IV. The bands from calculation III are in solid lines while those from calculation IV are in dashed lines. The occupied energies for both calculations (III and IV) are the same, as clearly shown by the perfect superposition of the valence bands from calculations III and IV. The fact that calculation V produced the same occupied energies signified that the ground state had been reached. Our work on GaP showed that the two consecutive calculations can produce the same occupied energies—as local minima—while the next calculation with an augmented basis sets further lowered some occupied energies. Among the three (3) calculations, the first had the smallest basis set called the *optimal basis set*.

Table 3 shows the electronic energies, obtained with the *optimal basis set* of calculation III, at high symmetry points in the Brillouin zone. The contents of this table lend themselves to comparison with results from future experimental findings such as the UV and X-ray spectroscopy experiments.

**Figure 2:**
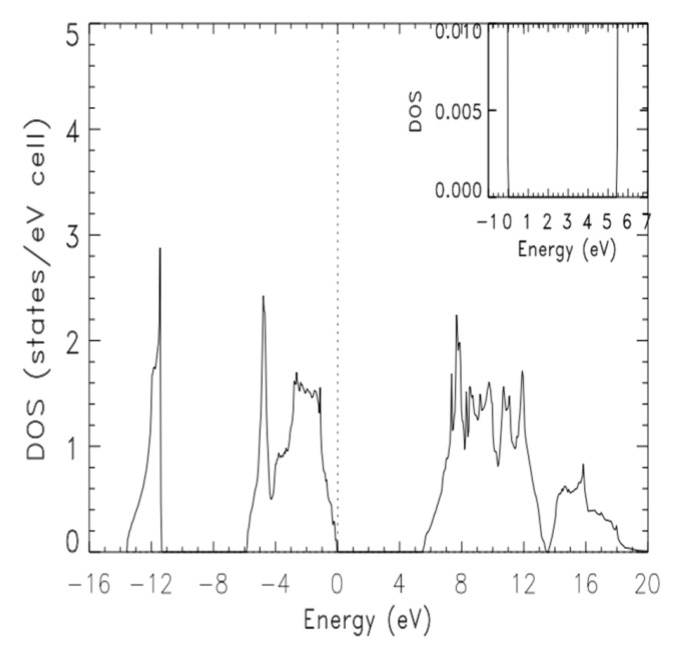
The total density of states (DOS) of zb-BeS, derived from the ground state band structure from calculation III.

**Figure 3:**
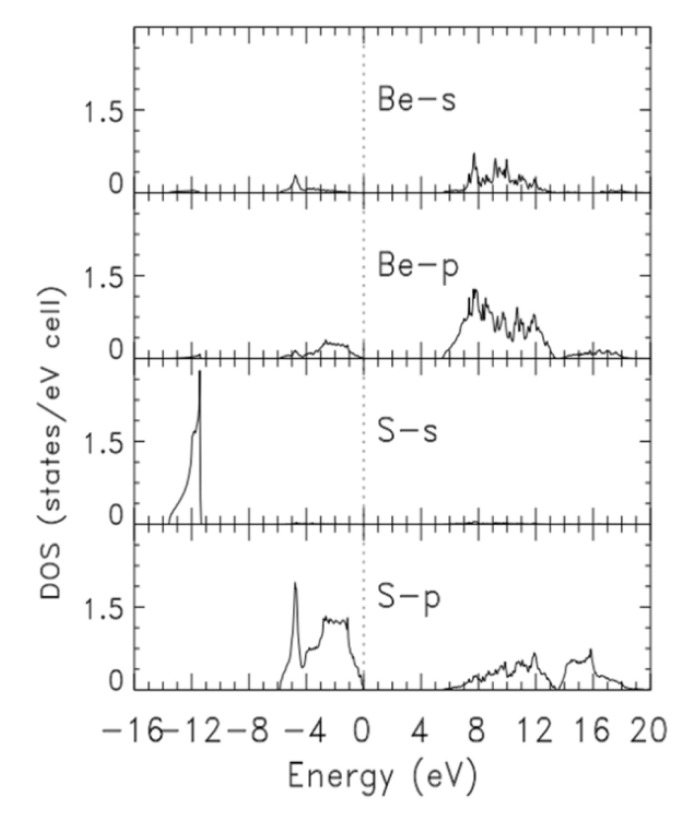
Calculated partial densities of states (pDOS) of zb-BeS derived from the ground state band structure from calculation III.

The calculated total density (DOS) and partial (pDOS) densities of states, are derived from the ground state band structure from calculation III. Figure 2 and Figure 3 show the DOS and pDOS, respectively, in the energy range of −16 eV to +20 eV. In both figures, the dashed, vertical line indicate the position of the Fermi level. While band widths and gaps can be estimated using Figure 2, their accurate values can be easily read from Table 2. The total valence band width and that of the lowest laying valence band were 13.585 eV and 2146 eV, respectively. The gap between the lowest laying band and the group of upper valence bands was 5.561 eV. We already noted the indirect band gap of 5.436 between the top of the valence band and the bottom of the conduction band. The width of the upper group of valence bands was 5.878 eV. As shown in Figure 3, the lowermost valence band is largely made up of S, with a faint contribution from Be s. The S p states contribute to the most to the upper group of valence bands, with relative small and tiny contributions from Be p and Be s, respectively. While Be p dominated at the very bottom of the conduction bands, the contributions of S p and Be s increased with energy, up to 12 eV.

### 3.2. Transport Properties

The determination of numerous material transport properties requires the use of effective masses. Electrical conductivity and mobility are two such quantities. We calculated electron effective masses around the minimum of the conduction band, while the heavy and light hole effective masses were calculated around the top of the valence band at the Γ point. We considered the (1,0,0), (1,1,0), and (1,1,1) directions for the hole effective masses. Our calculated effective masses are presented in part (a) for the electron and part (b) for the hole effective masses. The calculation performed by D.J Skutel [10], using a nonrelativistic formalism and slater’s free electron-exchange approximation, found the heavy hole and light hole at Γ in the (1,0,0) directions as 0.7 m_0_ and 0.4 m_0_, respectively, where m_0_ is the free electron mass. In (1,1,1), he found the effective masses for the heavy and light hole to be 1.7 m_0_ and 0.3 m_0_, respectively. Skutel also calculated the electron effective mass at the conduction band minimum, the (1,0,0) direction, as 1.0. This value is close to a third larger than our finding of 0.743.

As in the case of the electron effective mass in the (1,0,0) directions, the calculated hole effective masses from Skutel (10) were larger than the corresponding ones in part (b) of Table 4.

### 3.3. Structural Properties

**Figure 4:**
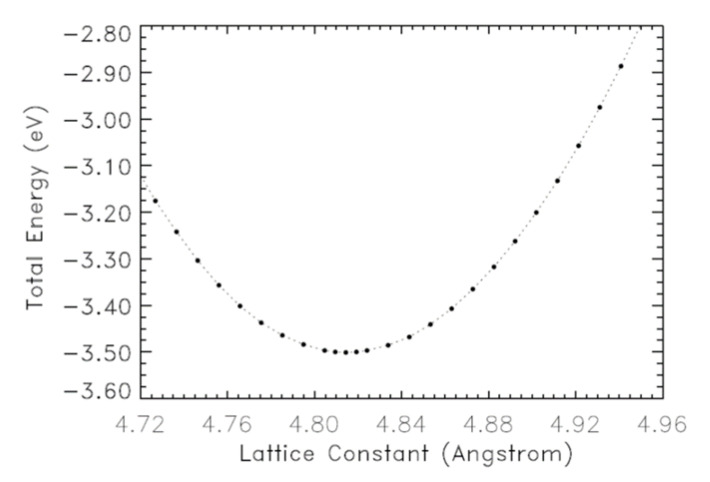
The total energy versus the lattice constant for zb-BeS.

The equilibrium lattice constant was determined to correspond to the minimum in the curve of the total energy versus the lattice constant, as shown in Figure 4. This predicted lattice constant was 4.814 Å for zb-BeS. This value is in good agreement with the first principle calculation finding of Okoye [17]. Our theoretical bulk modulus was 107.7 GPa. We compare our results to other theoretical calculations and experiments in Table 5**.**

## 4. Discussion

In this paper, we investigated the electronic, transport, and structural properties of beryllium sulfide in the zinc-blende phase, using the BZW-EF computational method and a local density approximation potential. Our study yielded an indirect band gap of 5.436 eV, with the minimum of the conduction band wis located at a point between Γ and the X point. The band structure shows that the minimum of the conduction band was located not at the X-point (where Eg = 6.5 eV) but shifted toward the Γ-point by 35% (by 0.35). The comprehensive description of our method [23,25] revealed a key distinction between BZW-EF calculations and other DFT calculations. We performed a generalized minimization of the energy in our self-consistent calculation to reach the ground state of the material, unlike other DFT calculations that employed a single basis set. As explained by Bagayoko [23], a single basis leads to a stationary state upon the attainment of self-consistency. These is an infinite number of such self-consistent results called stationary solutions. One cannot take any one of them arbitrarily to correspond to the ground state of the material. In these one basis set calculations, that basis set is generally selected to be quite large, in order to ensure completeness.

As explained by Bagayoko [25], when such a large basis set contains the optimal one, it leads to the ground state energies and to some unoccupied energies that are spuriously low by virtue of the Rayleigh theorem [30]. Specifically, these spurious, unoccupied energies are smaller than the corresponding unoccupied energies produced with the *optimal basis set*. As noted at the end of the presentation of our method, the referenced spurious lowering of unoccupied energies, with basis sets that are over-complete for the description of the ground state, is a plausible explanation of the general underestimation of the band gap by single basis set calculations such as the ones in Table 1. We should reiterate that the second theorem of DFT requires the generalized minimization of the energy functional to reach the ground state. As successively augmented basis sets produce energy functional that are lower for larger basis sets up the optimal one, the BZW-EF performs a generalized minimization of the energy far beyond any minimization that may result from self-consistent iterations with a single basis set. The above points are the reason our computational results possess the full, physical content of DFT and agree with the corresponding experimental ones, as is the case of the band gap of zb-BeS, for which we had a measured value around 5.5 eV. The theoretical bulk modulus from our work, 107.7 GPa, was about the same as the experimental result of 105 GPa of Narayana et al. [32].

## 5. Conclusions

By employing the BZW-EF computational method and a local density approximation potential, we studied the electronic, structural, and transport properties of the semiconductor BeS, in the zinc-blende phase. Our results for the first principle self-consistent calculations of the material and conclusion are summarized below. Our results possess the full, physical content of DFT by virtue of our generalized minimization of the energy functional (a) to reach the ground state while (b) avoiding over-complete basis sets. The electronic structure calculations showed that zb-BeS has an indirect band gap of 5.436 eV that agrees well with the available experimental value around 5.5 eV. Over 15 previous ab-initio LDA and GGA calculations uniformly underestimated this band gap by more than 50% in some cases, as per the contents in Table 1. As was the case in several previous studies by our group, some of which are listed below as references, future experiments are expected to confirm our results for which we could not find corresponding experimental ones. This assertion is expected to hold not only for the equilibrium lattice constant (4.814 Å), but also for the effective masses and the widths and other features of the band structures and related densities of states.

## Figures and Tables

**Table 1:** Previous calculated indirect band gap of zb-BeS using various computational techniques and potentials.

Computational Technique	Potential	Band Gap, Eg (eV)
Pseudopotential Plane Wave (PP-PW)	LDA	2.911 [5]
PP-PW	LDA	2.75 [6]
PP-PW	LDA	2.816 [8]
Empirical Pseudopotential Method	LDA	2.38 [9]
Orthogonalized Plane Wave	LDA	4.17 [10]
Full Potential Linearized Augmented Plane Wave (FP-LAPW)	LDA	2.828 [11]
PP-PW	LDA	2.912 [12]
Augmented plane Waves plus Local orbitals (APW + lo)	LDA	2.78 [13]
PP-PW	LDA	2.83 [14]
FP-LAPW	LSDA	2.847 [7]
PP-PW	GGA	3.041 [5]
PP-PW	GGA	3.7 [15]
PP-PW	GGA	3.192 [12]
PP-PW	GGA	3.11 [16]
FP-LAPW	GGA	3.141 [11]
FP-LAPW	GGA	3.13 [17]
Plane Wave pseudopotential (PW-PP)	GGA	3.15 [18]
APW + lo	Perdew-Burke-Erzerhof (PBE-GGA)	3.10 [13]
PP-PW	Sx-LDA	4.071 [12]
FP-LAPW	EV-GGA	4.241 [11]
Augmented Plane Wave	Hartee-Fock(HF)	6.10 [19]
PW-PP	Quansiparticle Self-Consistent Green’s Function (QPscGW)	5.27 [18]
PW-PP	Green’s Function (G_o_W_o_)	4.62 [18]
PP-PW	G_o_W_o_	4.45 [16]
Experiment using optical absorption measurement on the BeS platelets		>5.5 [4]

**Table 2:** The successive, self-consistent calculations for zinc-blende beryllium sulfide, using the BZW-EF computational approach. We performed our calculation utilizing the experimental lattice constant of 4.86 Å.

Calculation Number	Beryllium (Be^2+^)	Sulfur (S^2−^) (1s^2^2s^2^2p^6^ in Core)	No of Valence Functions	Energy Gap (eV)
I	1s^2^2s^0^2p^0^	3s^2^3p^6^	18	7.486
II	1s^2^2s^0^2p^0^3p^0^	3s^2^3p^6^	24	6.345
III	1s^2^2s^0^2p^0^3p^0^	3s^2^3p^6^4p^0^	30	5.438
IV	1s^2^2s^0^2p^0^3p^0^3s^0^	3s^2^3p^6^4p^0^	32	5.406
V	1s^2^2s^0^2p^0^3p^0^3s^0^	3s^2^3p^6^4p^0^4s^0^	34	5.361

**Table 3:** Calculated, electronic energies (in eV) of zb-BeS, at high symmetry points in the Brillouin zone, obtained from calculation III, using the BZW-EF method, with an experimental room temperature lattice constant of 4.863 Å. The indirect band gap from Γ to X to 5.436 eV.

L-Point	Γ-Point	X-Point (1–0.35)	X-Point	K-Point
25.451	29.334	25.824	24.420	22.099
17.165	29.334	15.998	16.067	14.157
13.482	9.794	11.256	13.179	12.276
8.463	6.784	11.256	13.179	11.816
8.463	6.784	10.196	7.564	10.040
7.593	6.784	5.436	6.505	7.207
−1.121	0.000	−2.215	−2.775	−2.299
−1.121	0.000	−2.215	−2.775	−4.162
−5.878	0.000	−3.810	−5.144	−4.639
−11.970	−13.585	−12.215	−11.424	−11.439

**Table 4:** Calculated electron (M_e_) and hole effective masses for zb-BeS, in the indicated directions, in (**a**) for M_e_ and (**b**) for heavy hole (M_hh_) and light hole (M_lh_) effective masses. The effective mass are in units of the free electron mass (m_0_).

(a) M_e_ (X-Γ) Longitudinal		M_e_ (X-U) Transverse		M_e_ (X-W) Transverse	
0.743		0.317		0.313	
**(b) (Γ-L) in (1,1,1) Direction**		**(Γ-X) in (1,0,0) Direction**		**(Γ-K) in (1,1,0) Direction**	
Mhh	Mlh	Mhh	Mlh	Mhh	Mlh
1.295	0.216	0.585	0.381	0.803	0.285

**Table 5:** Calculated lattice constant (a_o_) and bulk modulus (B) utilizing the LDA potential compared to the experiment and other theoretical calculations.

Potential	a_o_ (Å)	B	References
LDA	4.814	107.7	Present
LDA	4.773	101.9	Theory [8]
LDA	4.81	93	Theory [14]
LDA	4.745	116	Theory [6]
LDA	4.800	102	Theory [13]
GGA	4.887	92	Theory [17]
PBE-GGA	4.878	93	Theory [13]
Experiment	4.870	105	Exp [32]

## Data Availability

The data presented in this study are available on request from the corresponding author.

## References

[1] Landwehr G. (1998). Molecular-beam epitaxy of beryllium-chalcogenide-based thin films and quantum-well structures. J. Appl. Phys..

[2] Ivanov S.V., Toropov A.A., Sorokin S.V., Shubina T.V., Il’inskaya N.D., Lebedev A.V., Sedova I.V., Kop’ev P.S., Alferov Z.I., Lugauer H.J. (1998). Molecular beam epitaxy of alternating-strain ZnSe-based multilayer heterostructures for blue-green lasers. Semiconductors.

[3] Muñoz A., Rodríguez-Hernández P., Mujica A. (1996). Ground-state properties and high-pressure phase of beryllium chalcogenides BeSe, BeTe, and BeS. Phys. Rev. B Condens. Matter Mater. Phys..

[4] Yim W., Dismukes P., Stofko J., Paff J. (1972). Synthesis and some properties of BeTe, BeSe and BeS. J. Phys. Chem. Solids.

[5] Elias B. (2013). Theoretical investigation of the structural, electronic, elastic, and optical properties of zinc- blende bes under high pressure. Theor. Investig..

[6] Gonzalez-Diaz M., Rodriguez-Hernandez P., Munoz A. (1996). Elastic constants and electronic structure of beryllium chalcogenides BeS, BeSe, and BeTe from first-principles calculations. Phys. Rev. B.

[7] Benosman N., Amrane N., Méçabih S., Aourag H. (2001). Structural and electronic properties of bulk BeS. Phys. B Condens. Matter.

[8] Van Camp P.E. (1996). Ground state properties and structural phase transformation of beryllium sulphide, solid state communication. J. Phys. Condens. Matter.

[9] Van Vechten J.A. (1969). Quantum dielectric theory of electronegativity in covalent systems. II. Ionization potentials and interband transition energies. Phys. Rev..

[10] Skutel D.J. (1970). Energy band structure of BeS, BeSe, and BeTe. Phys. Rev..

[11] Al-Douri Y., Baaziz H., Charifi Z., Reshak A.H. (2012). Density functional study of optical properties of beryllium chalcogenides compounds in nickel arsenide B8 structure. Phys. B Condens. Matter.

[12] Guo L., Hu G., Zhang S., Feng W., Zhang Z. (2013). Structural, elastic, electronic and optical properties of beryllium chalcogenides BeX (X = S, Se, Te) with zinc-blende structure. J. Alloys Compd..

[13] Heciri D., Beldi L., Drablia S., Meradji H., Derradji N.E., Belkhir H., Bouhafs B. (2007). First-principles elastic constants and electronic structure of beryllium chalcogenides BeS, BeSe and BeTe. Comput. Mater. Sci..

[14] Srivastava G.P., Tütüncü H.M., Günhan N. (2004). First-principles studies of structural, electronic, and dynamical properties of Be chalcogenides. Phys. Rev. B Condens. Matter Mater. Phys..

[15] Faraji S., Mokhtari A. (2010). Ab initio study of the stability and electronic properties of wurtzite and zinc-blende BeS nanowires. Phys. Lett. Sect. A Gen. At. Solid State Phys..

[16] Yadav P.S., Yadav R.K., Agrawal S., Agrawal B.K. (2007). Ab initio study of electronic and optical properties of Be-chalcogenides in GW approximation. Phys. E Low-Dimens. Syst. Nanostructures.

[17] Okoye C.M.I. (2004). Structural, electronic, and optical properties of beryllium monochalcogenides. Eur. Phys. J. B.

[18] Wang K.L., Gao S.P. (2018). Phonon dispersions, band structures, and dielectric functions of BeO and BeS polymorphs. J. Phys. Chem. Solids.

[19] Sarkar R.L., Chatterjee S. (1977). Electronic energy bands of BeS, BeSe and BeTe. J. Phys. C Solid State Phys..

[20] Alder B.J., Ceperley D. (1980). Ground State of the Electron Gas by a Stochastic Method. Phys. Rev. Lett..

[21] Vosko S., Wilk L., Nusair M. (1980). Accurate spin-dependent electron liquid correlation energies for local spin density calculations: A critical analysis. Can. J. Phys..

[22] Bagayoko D., Zhao G.L., Fan J.D., Wang J.T. (1998). Ab initio calculations of the electronic structure and optical properties of ferroelectric tetragonal BaTiO3. J. Phys. Condens. Matter.

[23] Franklin L., Ekuma C., Zhao G., Bagayoko D. (2013). Bagayoko Density functional theory description of electronic properties of wurtzite zinc oxide. J. Phys. Chem. Solids.

[24] Bagayoko D., Franklin L., Zhao G.L. (2004). Predictions of electronic, structural, and elastic properties of cubic InN. J. Appl. Phys..

[25] Bagayoko D. (2014). Understanding density functional theory (DFT) and completing it in practice. AIP Adv..

[26] Ayirizia B.A., Malozovsky Y., Franklin L., Bhandari U., Bagayoko D. (2020). Ab-Initio Self-Consistent Density Functional Theory Description of Rock-Salt Magnesium Selenide (MgSe). Mater. Sci. Appl..

[27] Bagayoko D. (2016). Understanding the Relativistic Generalization of Density Functional Theory (DFT) and Completing It in Practice. J. Mod. Phys..

[28] Bhandari U., Ayirizia B.A., Malozovsky Y., Franklin L., Bagayoko D. (2020). First principle investigation of electronic, transport, and bulk properties of zinc-blende magnesium sulfide. Electronics.

[29] Feibelman P.J., Appelbaum J.A., Hamann D.R. (1979). Electronic structure of a Ti(0001) film. Phys. Rev. B.

[30] Harmon B.N., Weber W., Hamann D.R. (1982). Total-energy calculations for Si with a first-principles linear-combination-of-atomic-orbitals method. Phys. Rev. B.

[31] Ekuma C.E., Bagayoko D. (2011). Ab-initio electronic and structural properties of rutile titanium dioxide. Jpn. J. Appl. Phys..

[32] Narayana C., Nesamony V., Ruoff A. (1997). Phase transformation of BeS and equation-of-state studies to 96 GPa. Phys. Rev. B Condens. Matter Mater. Phys..

